# Development and validation of a clinical nomogram based on lasso-logistic regression for predicting prostate cancer with PSA 4-20.0 ng/mL: a retrospective study

**DOI:** 10.3389/fendo.2026.1757255

**Published:** 2026-03-26

**Authors:** Mengling Ying, Lijun Wang, Mengge Yang, Mang Ke, Liangxue Sun

**Affiliations:** 1 Department of Urology, The People’s Hospital of Yuhuan, Yuhuan, Zhejiang, China; 2 Department of Urology, Taizhou Hospital of Zhejiang Province Affiliated to Wenzhou Medical University, Enze Hospital, Taizhou Enze Medical Center (Group), Taizhou, Zhejiang, China; 3 Department of Emergency, Taizhou Hospital of Zhejiang Province Affiliated to Wenzhou Medical University, Enze Hospital, Taizhou Enze Medical Center (Group), Taizhou, Zhejiang, China; 4 Department of Urology, Taizhou Hospital of Zhejiang Province Affiliatedo Wenzhou Medical University, Linhai, Zhejiang, China

**Keywords:** LASSO, nomogram, PI-RADS, prostate cancer, prostate health index

## Abstract

**Background:**

Given the established diagnostic utility of the prostate health index (PHI) in prostate cancer (PCa), this study sought to incorporate PHI into a clinically applicable prediction model alongside conventional parameters, with the goal of refining biopsy selection in men presenting with PSA values of 4–20 ng/mL.

**Methods:**

We retrospectively collected clinical data from patients undergoing prostate biopsy at tertiary medical centers in China. Candidate variables were screened using least absolute shrinkage and selection operator (LASSO) regression, and the selected predictors were incorporated into a multivariable logistic regression model, which was subsequently presented as a nomogram. Model performance was evaluated in both the training and validation cohorts in terms of discrimination, calibration, and clinical utility.

**Results:**

A total of 314 patients were included, with 219 assigned to the training cohort and 95 to the validation cohort. LASSO regression identified prostate volume, blood glucose, low-density lipoprotein, triglycerides, urinary leukocyte count, hypertension, Prostate Imaging–Reporting and Data System score, platelet-to-lymphocyte ratio, albumin, the fPSA/tPSA ratio, and PHI as candidate variables. Multivariate analysis demonstrated that triglycerides, PI-RADS score, ALB, and PHI were independent predictors of PCa. The nomogram achieved good discriminatory performance, with an area under the receiver operating characteristic curve of 0.75 in the training cohort. Calibration curves and the *Hosmer–Lemeshow* test indicated good agreement between predicted and observed outcomes. Consistent performance was observed in the validation cohort.

**Conclusions:**

Our findings suggest that integrating clinical parameters into a PHI-based model can enhance the stratification of prostate cancer risk, potentially reducing unnecessary biopsies and improving patient outcomes.

## Introduction

Prostate cancer (PCa) is a common malignancy in men worldwide, with high incidence in Western countries and a rapidly growing burden in China (1). Early detection relies primarily on digital rectal examination (DRE) and prostate-specific antigen (PSA) testing; however, while PSA screening has improved detection rates, its limited specificity and high sensitivity often lead to unnecessary biopsies (2, 3). Notably, only about one quarter of patients with PSA levels of 4–10 ng/mL are ultimately diagnosed with PCa, and detection rates are comparable between patients with PSA levels of 4–10 and 10–20 ng/mL (4–6). Given these limitations, multiparametric biomarkers such as the Prostate Health Index (PHI), which integrates total PSA, free PSA, and [-2]proPSA, have emerged as superior predictors of clinically significant PCa (csPCa), particularly in men with PSA levels of 4–20 ng/mL, by improving risk stratification and reducing unnecessary biopsie (7, 8). Nevertheless, PHI alone may be insufficient for optimal clinical decision-making, as it does not consider additional clinical, imaging, or systemic factors that can influence PCa risk (9).

To address these limitations, we sought to develop an enhanced predictive model based on PHI that incorporates additional clinical and demographic parameters to improve diagnostic accuracy. Multiparametric magnetic resonance imaging (mpMRI) is now widely adopted as an initial imaging approach for prostate cancer detection and risk stratification (10). The Prostate Imaging Reporting and Data System (PI-RADS), derived from mpMRI, provides a standardized framework for lesion assessment and identification of suspicious prostate lesions (7). When combined with serological markers such as PSA, mpMRI, and PI-RADS have been shown to significantly improve early diagnostic precision (11, 12). Additionally, routine laboratory tests, including complete blood count–derived parameters, have been reported to provide supplementary value in prostate cancer diagnosis and risk stratification (13, 14). These integrative models enable more precise patient selection for targeted biopsy and enhance the detection of clinically significant prostate cancer (12). Beyond PSA derivatives and imaging biomarkers, emerging evidence suggests that metabolic and nutritional factors may also contribute to prostate cancer risk assessment. Metabolic parameters such as serum triglyceride levels have been associated with prostate cancer risk and tumor aggressiveness, potentially reflecting underlying metabolic dysregulation and cancer-associated lipid metabolism (15–17). Similarly, serum albumin, an indicator of nutritional status and systemic inflammation, has been associated with cancer prognosis and may provide insight into host–tumor interactions relevant to disease progression (18). Collectively, these findings indicate that metabolic and laboratory parameters may provide complementary information to traditional prostate-specific biomarkers and imaging features when incorporated into multivariable predictive models.

In recent prostate cancer research, penalized regression approaches such as LASSO have been widely used to select relevant clinical and imaging predictors and to construct robust logistic regression-based predictive models. A LASSO-regularized logistic regression model demonstrated good discrimination and clinical utility in predicting Gleason grade group upgrading among PCa patients, outperforming conventional logistic and other machine learning models (19). Similarly, a clinical prediction model developed using LASSO feature selection and multivariable logistic regression provided excellent risk stratification between benign and malignant conditions in patients with elevated PSA levels (20). Moreover, radiomics studies combining LASSO selected features with clinical parameters have also shown strong predictive performance in individualized prostate cancer risk models (21).

We developed and validated a PHI-based model to guide diagnostic decisions in patients with PSA levels of 4–20 ng/mL while reducing unnecessary interventions.

## Materials and methods

### Study design and patients selection

**Figure 1:**
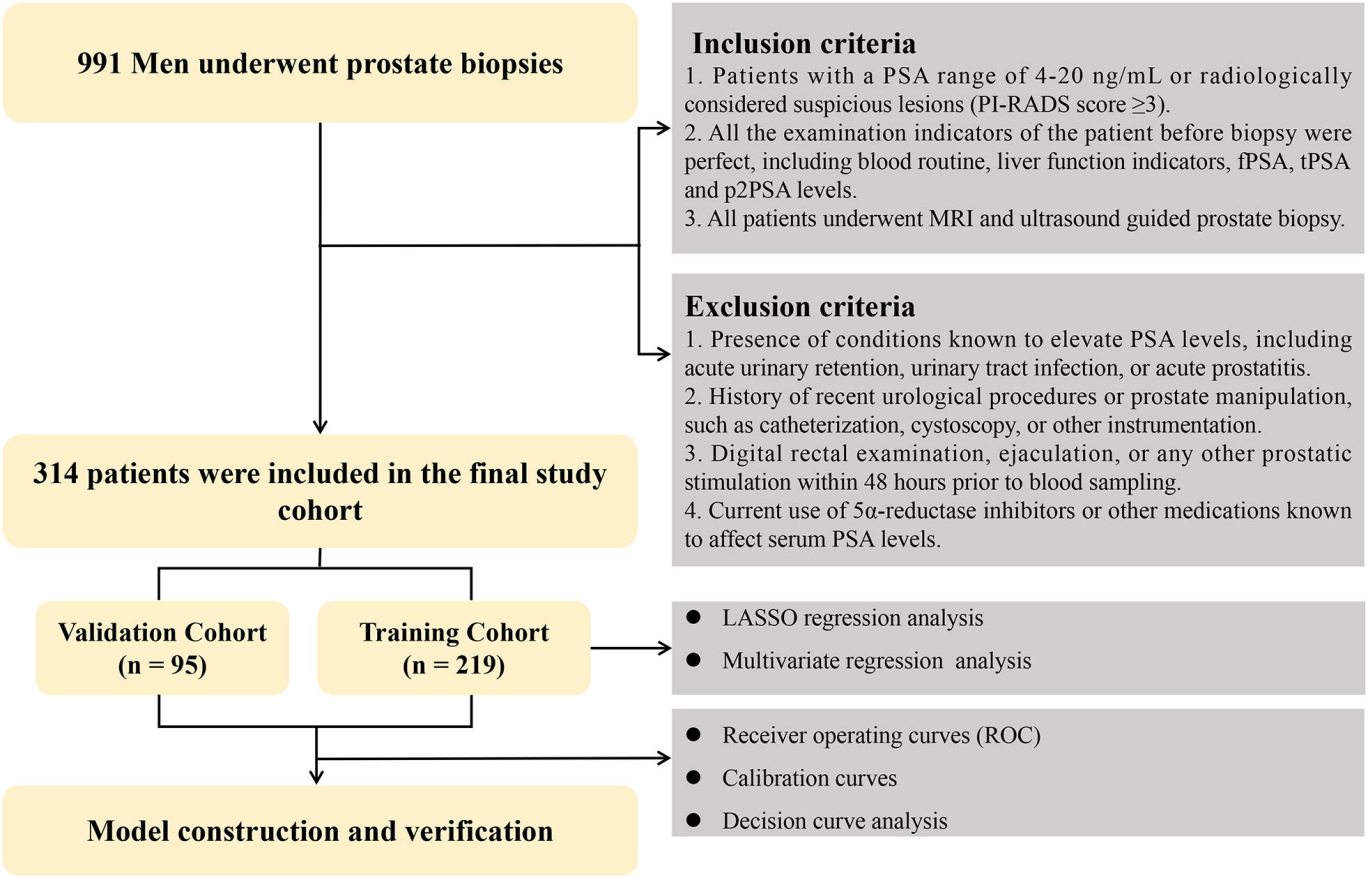
Flowchart illustrating the patient recruitment and selection process. A total of 991 patients undergoing prostate biopsy between January 2022 and October 2023 were retrospectively screened. Patients were included if they had PSA levels between 4 and 20 ng/mL or suspicious lesions on mpMRI (PI-RADS ≥ 3) and complete pre-biopsy clinical and laboratory data. Patients were excluded due to incomplete data or not meeting inclusion criteria. The final cohort was randomly divided into training and validation sets at a 7:3 ratio.

Following institutional approval, we retrospectively reviewed clinical records of patients who underwent prostate biopsy at our center between January 2022 and October 2023, with data extracted from the electronic medical record system. Eligible patients met following criteria: (1) serum PSA levels ranging from 4 to 20 ng/mL and/or radiologically suspicious lesions on multiparametric MRI (PI-RADS score ≥3); (2) availability of complete pre-biopsy laboratory assessments, including routine blood tests, liver function indices, total PSA (tPSA), free PSA (fPSA), and p2PSA; and (3) completion of MRI- and ultrasound guided prostate biopsy. Patients were excluded if they had conditions or recent procedures that could potentially influence serum PSA levels, including acute urinary retention, urinary tract infection, acute prostatitis, recent catheterization, cystoscopy, or prostate manipulation. In addition, patients who had undergone digital rectal examination, ejaculation, or other prostatic stimulation within 48 hours prior to blood sampling were excluded. Patients receiving 5α-reductase inhibitors or other medications known to affect PSA levels were also excluded. The study flowchart is presented in **Figure 1**.

### Biopsy protocol

Prostate biopsies were performed in accordance with standardized clinical guidelines to ensure procedural consistency. All patients underwent transperineal prostate biopsy. The procedure included routine preoperative evaluation, prophylactic antibiotic administration, and positioning in either the lithotomy or supine position. Under transrectal ultrasound guidance, biopsy needles were inserted through the perineal skin into the prostate, and multiple tissue cores were obtained from different regions to ensure comprehensive pathological assessment.

### Data collection and clinical variables

Demographic characteristics and laboratory data were retrospectively extracted from the hospital information system. Collected variables included (1): baseline clinical characteristics (2); serum biomarkers obtained within 24 hours prior to biopsy, including total cholesterol, high-density lipoprotein (HDL), low-density lipoprotein (LDL), triglycerides, neutrophil-to-lymphocyte ratio (NLR), platelet-to-lymphocyte ratio (PLR), absolute lymphocyte count (ALC), albumin (ALB), prognostic nutritional index (PNI), tPSA, fPSA/tPSA ratio, and prostate health index (PHI); and (3) prostate volume, urinary leukocyte and nitrite status, and comorbidities including hypertension, diabetes, and hyperuricemia. Prostate volume was calculated using the ellipsoid formula (width × height × length × 0.52). NLR and PLR were defined as the ratios of neutrophils to lymphocytes and platelets to lymphocytes, respectively. PNI was calculated as: PNI = albumin (g/L) + 5 × total lymphocyte count (10^9^/L). PHI was calculated using the formula: PHI = ((−2)proPSA/fPSA)/√PSA, and %p2PSA was calculated as [(p2PSA pg/mL)/(fPSA ng/mL × 1000)] × 100 (22, 23). PI-RADS scores were assigned according to Prostate Imaging–Reporting and Data System version 2.1 (PI-RADS v2.1) criteria (24). All biopsy specimens were reviewed by an experienced uropathologist, and prostate cancer was defined according to the 2014 International Society of Urological Pathology criteria as a Gleason score ≥ 3 + 3 (25, 26), while clinically significant prostate cancer (csPCa) was defined as a Gleason score of ≥ 3 + 4 (27).

### Mathematical framework and model construction

The prediction model was developed within a multivariable logistic regression framework to model the binary outcome of prostate cancer detection at biopsy. All candidate clinical and laboratory predictors were initially included in the analysis. To address potential multicollinearity and reduce the risk of overfitting, feature selection and coefficient shrinkage were performed using the least absolute shrinkage and selection operator (LASSO). The LASSO penalty parameter (λ) was determined using ten-fold cross-validation, with the value selected according to the standard “one standard error” rule (lambda.1se), which chooses the largest λ within one standard error of the minimum cross-validated −2 log-likelihood. This approach provides a more parsimonious model while maintaining predictive performance, as recommended in prior studies (28, 29). For completeness, we verified that the model obtained using the λ minimizing the cross-validated error (lambda.min) yielded broadly similar predictors, but the lambda.1se model was chosen for its interpretability and reduced risk of overfitting. Predictors with non-zero coefficients at the selected λ were subsequently entered into a standard multivariable logistic regression model to estimate odds ratios and construct the final prediction model. This modeling strategy follows established statistical methodology for LASSO-penalized logistic regression and is widely used in clinical prediction research (30, 31). Model discrimination was quantified by the area under the receiver operating characteristic curve (AUC) (32). Calibration performance was assessed using calibration plots and the Hosmer-Lemeshow goodness-of-fit test (33). Decision curve analysis (DCA) was conducted to evaluate the model’s clinical utility by calculating the net benefit at different probability thresholds (34). This modeling framework ensures transparent, reproducible, and clinically interpretable predictions without the need to include explicit mathematical equations in the main manuscript.

### Statistical analysis

All statistical computations were performed in R (version 4.2.2, https://www.R-project.org). We described and compared continuous variables using mean ± SD with *t-test* for normal distributions, and median (IQR) with *Mann-Whitney U test* for non-normal distributions. Categorical variables were reported as proportions and analyzed with the *Chi-square* test or *Fisher’s exact* test. Restricted cubic spline analysis was performed using the “rms” package, and the results were visualized using the “plotRCS” and “ggplot2” packages. Potential predictive features were selected via LASSO regression using the “glmnet” package, with the optimal value of λ determined through 10-fold cross-validation. A diagnostic nomogram was constructed using the “rms” package. Model discrimination and calibration were evaluated using ROC curves, calibration plots, and the Hosmer-Lemeshow goodness-of-fit test implemented in the “pROC” and “ResourceSelection” packages, respectively. Clinical utility was assessed using decision curve analysis. Statistical significance was accepted at P < 0.05.

## Results

### Demographic and clinical characteristics of the study population

**Figure 2:**
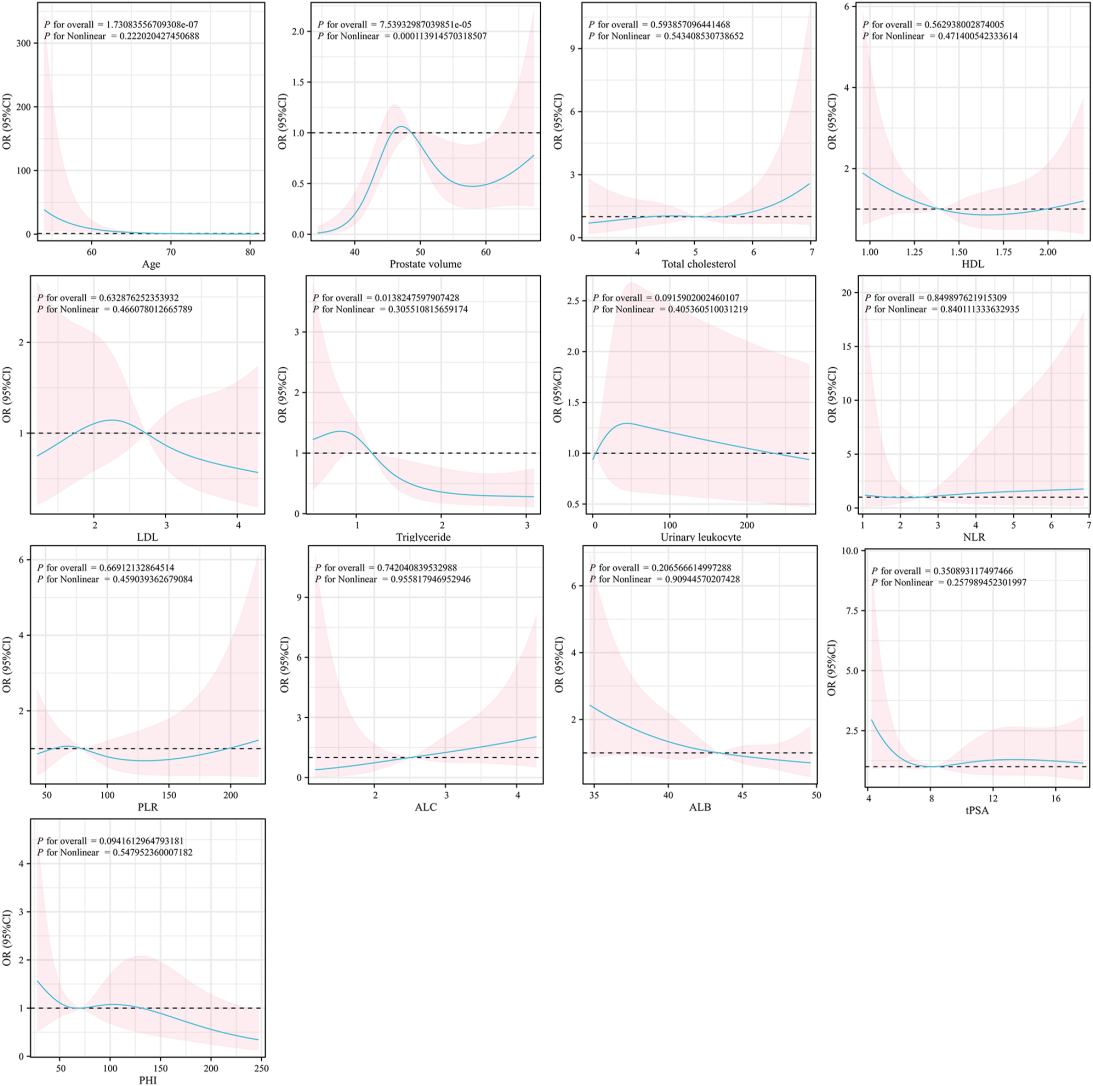
Restricted cubic spline (RCS) analysis depicting the associations between continuous clinical variables and prostate cancer risk. The y-axis represents the odds ratio (OR) with 95% confidence intervals (shaded areas), and the x-axis indicates the corresponding clinical variables. Reference values were set at the median of each variable. Solid lines indicate estimated ORs, while shaded areas denote 95% confidence intervals. P values for nonlinearity were calculated to assess potential nonlinear relationships.

After detailed screening, we included 314 patients with elevated PSA levels (4–20 ng/mL) who underwent prostate biopsy in our cohort. **Table 1** presents the baseline characteristics of these patients, comprising 215 with negative biopsy results and 99 with positive results. Significant differences were observed between the two groups in LDL, fPSA/tPSA ratio, PHI, and PI-RADS (P < 0.05), while other characteristics showed no significant differences. Using a restricted cubic spline, we illustrated the relationship between odds ratios (OR) and various clinical variables in a logistic regression model (**Figure 2**). Restricted cubic spline analysis demonstrated a significant non-linear association between prostate volume and prostate cancer risk (P for nonlinearity < 0.001), suggesting that the effect of prostate volume on biopsy outcomes varies across different volume ranges. The cohort was randomly split into training and validation sets at a 7:3 ratio using computer-generated randomization. **Table 2** and **Table 3** summarize the characteristics of patients in these two cohorts.

**Table 1:** Demographic and clinical characteristics of all patients in the study population.

Variables	Total (n = 314)	Negative biopsy (n = 215)	PCa (n = 99)	P-value
Age (year), Mean ± SD	67.81 ± 7.56	67.81 ± 7.58	67.82 ± 7.55	0.992
Prostate volume (mL), M (Q_1_, Q_3_)	44.52 (31.17, 61.86)	46.44 (29.77, 63.81)	43.57 (33.01, 55.38)	0.491
Blood glucose (mmol/L), M (Q_1_, Q_3_)	5.47 (5.12, 5.97)	5.46 (5.12, 6.01)	5.50 (5.12, 5.92)	0.889
Total cholesterol (mmol/L), M (Q_1_, Q_3_)	5.04 (4.25, 5.69)	5.10 (4.29, 5.76)	4.90 (4.15, 5.58)	0.092
HDL (mmol/L), M (Q_1_, Q_3_)	1.39 (1.21, 1.64)	1.40 (1.21, 1.65)	1.36 (1.21, 1.63)	0.522
LDL (mmol/L), M (Q_1_, Q_3_)	2.73 (2.14, 3.22)	2.80 (2.26, 3.25)	2.56 (2.00, 3.09)	0.024
Triglyceride (mmol/L), M (Q_1_, Q_3_)	1.19 (0.84, 1.66)	1.17 (0.81, 1.56)	1.25 (0.88, 1.98)	0.088
Urinary leukocyte ( /HP), M (Q_1_, Q_3_)	4.00 (1.00, 13.00)	4.00 (1.00, 14.00)	4.00 (2.00, 10.50)	0.997
NLR, M (Q_1_, Q_3_)	2.52 (1.82, 3.52)	2.57 (1.82, 3.54)	2.49 (1.96, 3.48)	0.982
PLR, M (Q_1_, Q_3_)	79.25 (61.20, 114.47)	78.85 (61.92, 114.50)	83.82 (60.03, 114.42)	0.999
ALC ( /μL), M (Q_1_, Q_3_)	2.51 (2.00, 3.16)	2.50 (2.00, 3.17)	2.56 (2.00, 3.10)	0.929
ALB (g/L), M (Q_1_, Q_3_)	43.40 (41.00, 46.10)	43.30 (40.60, 45.95)	43.80 (41.70, 46.45)	0.147
PNI, M (Q_1_, Q_3_)	56.42 (52.05, 60.50)	56.00 (51.83, 60.27)	56.70 (53.00, 60.80)	0.41
tPSA (ng/mL), M (Q_1_, Q_3_)	8.21 (6.10, 11.29)	7.70 (5.94, 11.46)	8.53 (6.42, 10.87)	0.388
fPSA/tPSA ratio, M (Q_1_, Q_3_)	0.15 (0.10, 0.20)	0.16 (0.11, 0.21)	0.13 (0.09, 0.19)	0.002*
PHI, M (Q_1_, Q_3_)	71.05 (49.60, 104.40)	59.90 (46.30, 91.95)	96.00 (66.90, 130.50)	<0.001*
PI-RADS, n (%)				0.001*
1	9 (2.87)	6 (2.79)	3 (3.03)	
2	159 (50.64)	122 (56.74)	37 (37.37)	
3	102 (32.48)	68 (31.63)	34 (34.34)	
4	31 (9.87)	14 (6.51)	17 (17.17)	
5	13 (4.14)	5 (2.33)	8 (8.08)	
Urinary nitrite, n (%)				0.810
Negative	304 (96.82)	209 (97.21)	95 (95.96)	
Positive	10 (3.18)	6 (2.79)	4 (4.04)	
Hypertension, n (%)				0.181
No	216 (68.79)	153 (71.16)	63 (63.64)	
Yes	98 (31.21)	62 (28.84)	36 (36.36)	
Diabetes, n (%)				0.927
No	283 (90.13)	194 (90.23)	89 (89.90)	
Yes	31 (9.87)	21 (9.77)	10 (10.10)	
Hyperuricemia, n (%)				1
No	308 (98.09)	211 (98.14)	97 (97.98)	
Yes	6 (1.91)	4 (1.86)	2 (2.02)	

t, t-test; Z, Mann-Whitney test, χ², Chi-square test; SD, standard deviation; M, Median, Q_1_, 1st Quartile, Q_3_, 3st Quartile, PCa:Prostate Cancer; HDL, High density lipoprotein; LDH, Low density lipoprotein; NLR, Neutrophil to Lymphocyte Ratio; PLR, Platelet to Lymphocyte Ratio; ALC, Absolute Lymphocyte Count; ALB, Albumin; PNI, Prognostic nutritional index; PSA, Prostate-specific antigen; PHI, Prostate Health Index; PI-RADS, Prostate Imaging-Reporting and Data System. *Statistical difference.

**Table 2:** Patient characteristics in training and validation cohorts.

Variables	Training cohort (n = 219)	Validation cohort (n = 95)	P-value
Age (year), Mean ± SD	67.74 ± 7.64	67.97 ± 7.41	0.810
Prostate volume (mL), M (Q_1_, Q_3_)	46.68 (32.31, 62.96)	39.75 (26.37, 55.79)	0.053
Blood glucose (mmol/L), M (Q_1_, Q_3_)	5.44 (5.11, 5.97)	5.59 (5.22, 5.98)	0.271
Total cholesterol (mmol/L), M (Q_1_, Q_3_)	4.96 (4.24, 5.62)	5.26 (4.32, 5.86)	0.058
HDL (mmol/L), M (Q_1_, Q_3_)	1.38 (1.21, 1.63)	1.40 (1.21, 1.67)	0.560
LDL (mmol/L), M (Q_1_, Q_3_)	2.66 (2.12, 3.17)	2.82 (2.20, 3.27)	0.246
Triglyceride (mmol/L), M (Q_1_, Q_3_)	1.20 (0.83, 1.63)	1.17 (0.86, 1.67)	0.832
Urinary leukocyte ( /HP), M (Q_1_, Q_3_)	4.00 (2.00, 15.00)	3.00 (1.00, 10.00)	0.356
NLR, M (Q_1_, Q_3_)	2.64 (1.98, 3.54)	2.45 (1.71, 3.48)	0.189
PLR, M (Q_1_, Q_3_)	79.30 (60.94, 114.56)	79.20 (61.92, 113.19)	0.976
ALC ( /μL), M (Q_1_, Q_3_)	2.48 (2.00, 3.04)	2.59 (2.00, 3.29)	0.251
ALB (g/L), M (Q_1_, Q_3_)	43.50 (40.90, 46.10)	42.80 (41.00, 46.10)	0.756
PNI, M (Q_1_, Q_3_)	56.00 (52.02, 60.15)	56.60 (52.23, 61.10)	0.324
tPSA (ng/mL), M (Q_1_, Q_3_)	8.16 (5.99, 11.17)	8.36 (6.16, 11.38)	0.799
fPSA/tPSA ratio, M (Q_1_, Q_3_)	0.16 (0.11, 0.21)	0.13 (0.10, 0.17)	0.008*
PHI, M (Q_1_, Q_3_)	75.90 (53.05, 104.30)	66.00 (45.25, 107.65)	0.113
PI-RADS, n (%)			0.581
1	5 (2.28)	4 (4.21)	
2	115 (52.51)	44 (46.32)	
3	72 (32.88)	30 (31.58)	
4	19 (8.68)	12 (12.63)	
5	8 (3.65)	5 (5.26)	
Urinary nitrite, n (%)			0.286
Negative	210 (95.89)	94 (98.95)	
Positive	9 (4.11)	1 (1.05)	
Hypertension, n (%)			0.156
No	156 (71.23)	60 (63.16)	
Yes	63 (28.77)	35 (36.84)	
Diabetes, n (%)			0.798
No	198 (90.41)	85 (89.47)	
Yes	21 (9.59)	10 (10.53)	
Hyperuricemia, n (%)			0.777
No	214 (97.72)	94 (98.95)	
Yes	5 (2.28)	1 (1.05)	

t, t-test; Z, Mann-Whitney test, χ², Chi-square test; SD, standard deviation; M, Median, Q_1_, 1st Quartile, Q_3_, 3st Quartile, PCa, Prostate Cancer; HDL, High density lipoprotein; LDH, Low density lipoprotein; NLR, Neutrophil to Lymphocyte Ratio; PLR, Platelet to Lymphocyte Ratio; ALC, Absolute Lymphocyte Count; ALB, Albumin; PNI, Prognostic nutritional index; PSA, Prostate-specific antigen; PHI, Prostate Health Index; PI-RADS, Prostate Imaging-Reporting and Data System. *Statistical difference.

**Table 3:** Patient characteristics in training and validation cohorts with Negative biopsy and PCa.

Variables	Training cohort			Validation cohort		
	Negative biopsy (n = 147)	PCa (n = 72)	P-value	Negative biopsy (n = 68)	PCa (n = 27)	P-value
Age (year), Mean ± SD	67.60 ± 7.55	68.04 ± 7.86	0.688	68.26 ± 7.68	67.22 ± 6.75	0.539
Prostate volume (mL), M (Q_1_, Q_3_)	48.97 (31.37, 66.46)	45.11 (33.96, 56.73)	0.445	42.61 (26.05, 56.75)	39.04 (29.97, 50.15)	0.763
Blood glucose (mmol/L), M (Q_1_, Q_3_)	5.43 (5.11, 6.03)	5.46 (5.11, 5.89)	0.841	5.59 (5.23, 5.97)	5.57 (5.22, 5.96)	0.650
Total cholesterol (mmol/L), M (Q_1_, Q_3_)	5.06 (4.24, 5.64)	4.80 (4.26, 5.41)	0.282	5.29 (4.54, 5.91)	5.23 (4.00, 5.69)	0.188
HDL (mmol/L), M (Q_1_, Q_3_)	1.39 (1.21, 1.61)	1.35 (1.21, 1.63)	0.803	1.40 (1.25, 1.75)	1.40 (1.17, 1.54)	0.133
LDL (mmol/L), M (Q_1_, Q_3_)	2.74 (2.24, 3.24)	2.57 (2.04, 2.99)	0.152	2.92 (2.39, 3.29)	2.43 (1.87, 3.17)	0.082
Triglyceride (mmol/L), M (Q_1_, Q_3_)	1.18 (0.82, 1.58)	1.23 (0.85, 1.91)	0.289	1.15 (0.77, 1.49)	1.25 (1.10, 2.08)	0.132
Urinary leukocyte ( /HP), M (Q_1_, Q_3_)	4.00 (1.00, 17.00)	4.00 (2.00, 11.25)	0.914	4.00 (1.00, 10.50)	3.00 (1.00, 7.00)	0.784
NLR, M (Q_1_, Q_3_)	2.62 (1.90, 3.49)	2.69 (2.09, 3.56)	0.374	2.48 (1.77, 3.65)	2.17 (1.65, 2.81)	0.144
PLR, M (Q_1_, Q_3_)	78.64 (61.39, 113.87)	84.89 (60.82, 121.40)	0.598	80.65 (62.28, 119.38)	77.00 (54.69, 110.69)	0.393
ALC ( /μL), M (Q_1_, Q_3_)	2.49 (2.02, 3.15)	2.42 (1.95, 2.96)	0.385	2.50 (1.97, 3.23)	2.70 (2.18, 3.46)	0.168
ALB (g/L), M (Q_1_, Q_3_)	43.30 (40.55, 45.80)	44.00 (41.70, 46.50)	0.17	42.80 (40.72, 46.23)	43.30 (41.60, 45.60)	0.488
PNI, M (Q_1_, Q_3_)	55.65 (51.92, 60.27)	56.45 (52.05, 59.42)	0.921	56.55 (51.75, 60.16)	57.45 (55.55, 61.67)	0.176
tPSA (ng/mL), M (Q_1_, Q_3_)	8.16 (5.99, 11.50)	8.20 (6.04, 10.55)	0.935	7.10 (5.88, 11.46)	8.82 (7.82, 11.27)	0.112
fPSA/tPSA ratio, M (Q_1_, Q_3_)	0.17 (0.12, 0.21)	0.14 (0.09, 0.19)	0.022*	0.14 (0.10, 0.18)	0.11 (0.07, 0.13)	0.018*
PHI, M (Q_1_, Q_3_)	63.70 (48.55, 93.47)	94.95 (67.42, 117.90)	<0.001*	52.60 (39.32, 82.30)	102.10 (66.30, 142.25)	<0.001*
PI-RADS, n (%)			0.018*			0.010*
1	4 (2.72)	1 (1.39)		2 (2.94)	2 (7.41)	
2	84 (57.14)	31 (43.06)		38 (55.88)	6 (22.22)	
3	48 (32.65)	24 (33.33)		20 (29.41)	10 (37.04)	
4	9 (6.12)	10 (13.89)		5 (7.35)	7 (25.93)	
5	2 (1.36)	6 (8.33)		3 (4.41)	2 (7.41)	
Urinary nitrite, n (%)			1			0.284
Negative	141 (95.92)	69 (95.83)		68 (100.00)	26 (96.30)	
Positive	6 (4.08)	3 (4.17)		0 (0.00)	1 (3.70)	
Hypertension, n (%)			0.682			0.056
No	106 (72.11)	50 (69.44)		47 (69.12)	13 (48.15)	
Yes	41 (27.89)	22 (30.56)		21 (30.88)	14 (51.85)	
Diabetes, n (%)			0.352			0.219
No	131 (89.12)	67 (93.06)		63 (92.65)	22 (81.48)	
Yes	16 (10.88)	5 (6.94)		5 (7.35)	5 (18.52)	
Hyperuricemia, n (%)			1			1
No	144 (97.96)	70 (97.22)		67 (98.53)	27 (100.00)	
Yes	3 (2.04)	2 (2.78)		1 (1.47)	0 (0.00)	

t, t-test; Z, Mann-Whitney test, χ², Chi-square test, -, Fisher exact; SD, standard deviation; M, Median, Q_1_, 1st Quartile, Q_3_, 3st Quartile, PCa, Prostate Cancer; HDL, High density lipoprotein; LDH, Low density lipoprotein; NLR, Neutrophil to Lymphocyte Ratio; PLR, Platelet to Lymphocyte Ratio; ALC, Absolute Lymphocyte Count; ALB, Albumin; PNI, Prognostic nutritional index; PSA, Prostate-specific antigen; PHI, Prostate Health Index; PI-RADS, Prostate Imaging-Reporting and Data System. *Statistical difference.

### Prediction of risk factors based on Lasso-Logistic regression

**Figure 3:**
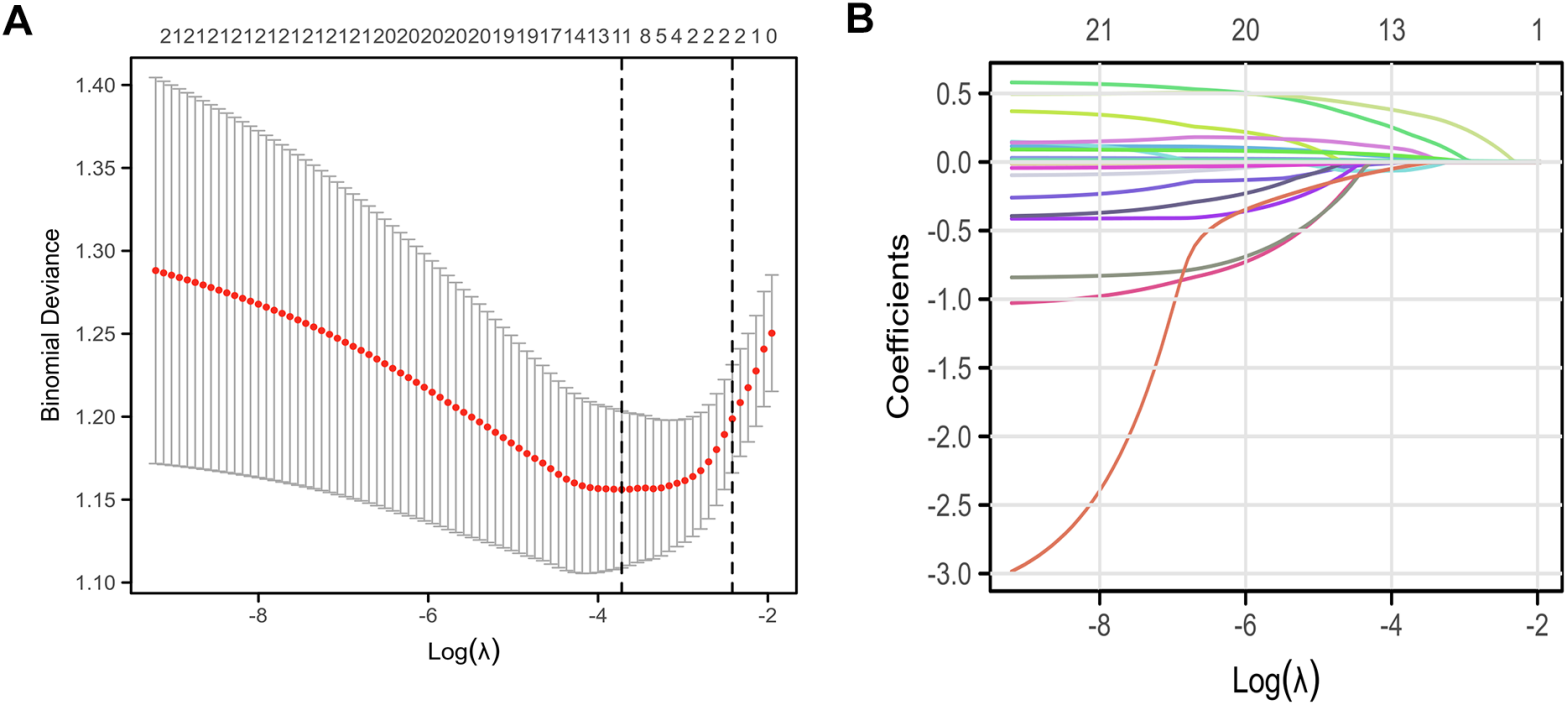
Variable selection using least absolute shrinkage and selection operator (LASSO) regression. **(A)** Ten-fold cross-validation for tuning parameter (λ) selection. The mean cross-validated −2 log-likelihood is plotted against log(λ). The vertical dotted lines indicate the λ values selected by two standard approaches: λ_min, which minimizes the cross-validated error, and λ_1se, which is the largest λ within one standard error of the minimum error. The horizontal grey bars represent the standard error of the cross-validated deviance. Numbers above the plot indicate the number of predictors retained at each λ. The λ_1se value was used to select predictors for inclusion in the final multivariable logistic regression model. **(B)** LASSO coefficient profiles of candidate predictors. Each curve represents the trajectory of a variable’s coefficient as the penalty parameter λ varies. Coefficients shrink toward zero as λ increases, illustrating how less informative variables are penalized and excluded from the model. The vertical dotted line corresponds to the λ_1se value selected by cross-validation. All LASSO analysis were performed in R version 4.2.2 using the “glmnet” package with all candidate clinical and laboratory variables from the training cohort.

We incorporated all variables into the analysis, screening risk factors with the LASSO regression algorithm, and selected the optimal λ value through 10-fold cross-validation (**Figures 3A, B**). The variables identified by lambda.1se included prostate volume, blood glucose, LDL, triglycerides, urinary leukocytes, hypertension, PI-RADS, PLR, ALB, fPSA/tPSA ratio, and PHI. From logistic regression analysis, we identified four clinical features to construct the prostate cancer diagnostic model. The multivariate analysis results included triglycerides (OR [95% CI]: 1.57 [1.05–2.34], P = 0.026), PI-RADS = 5 (OR [95% CI]: 22.14 [1.19–411.37], P = 0.038), ALB (OR [95% CI]:1.11 [1.02 ~ 1.20], P = 0.014), and PHI (OR [95% CI]: 1.01 [1.01–1.02], P < 0.001) (**Table 4**).

**Table 4:** Multivariate logistic regression analysis for clinical features based Lasso regression analysis.

Variables	Coefficient	S.E	P-value	OR (95%CI)
Triglyceride (mmol/L)	0.45	0.2	0.026*	1.57 (1.05 ~ 2.34)
PI-RADS				
1				1.00 (Reference)
2	1.19	1.25	0.341	3.29 (0.28 ~ 38.07)
3	1.37	1.26	0.278	3.92 (0.33 ~ 46.14)
4	2.13	1.33	0.108	8.41 (0.62 ~ 113.33)
5	3.1	1.49	0.038*	22.14 (1.19 ~ 411.37)
ALB (g/L)	0.1	0.04	0.014*	1.11 (1.02 ~ 1.20)
PHI	0.01	0	<0.001*	1.01 (1.01 ~ 1.02)

OR, Odds Ratio; CI, Confidence Interval; ALB, Albumin; PHI, Prostate Health Index; PI-RADS, Prostate Imaging-Reporting and Data System; S.E, Standard error; *Statistical difference.

### Performance and validation of the nomogram

**Figure 4:**
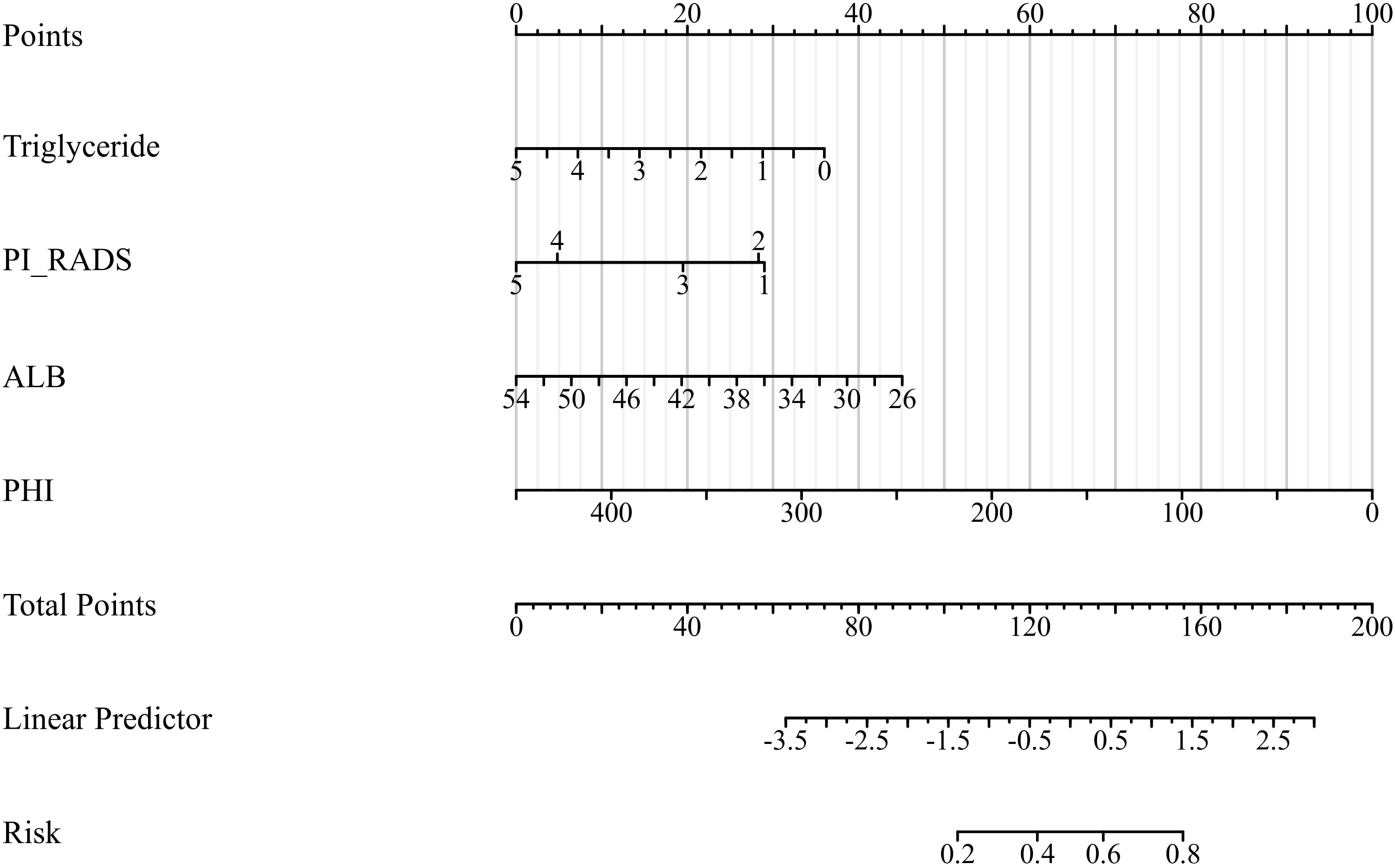
Nomogram for predicting the probability of prostate cancer in patients with PSA levels between 4 and 20 ng/mL. The nomogram integrates PI-RADS score, serum triglycerides, albumin (ALB), and prostate health index (PHI). Each predictor corresponds to a point value at the top scale; the sum of points projects to the estimated probability of prostate cancer on the bottom scale.

**Figure 5:**
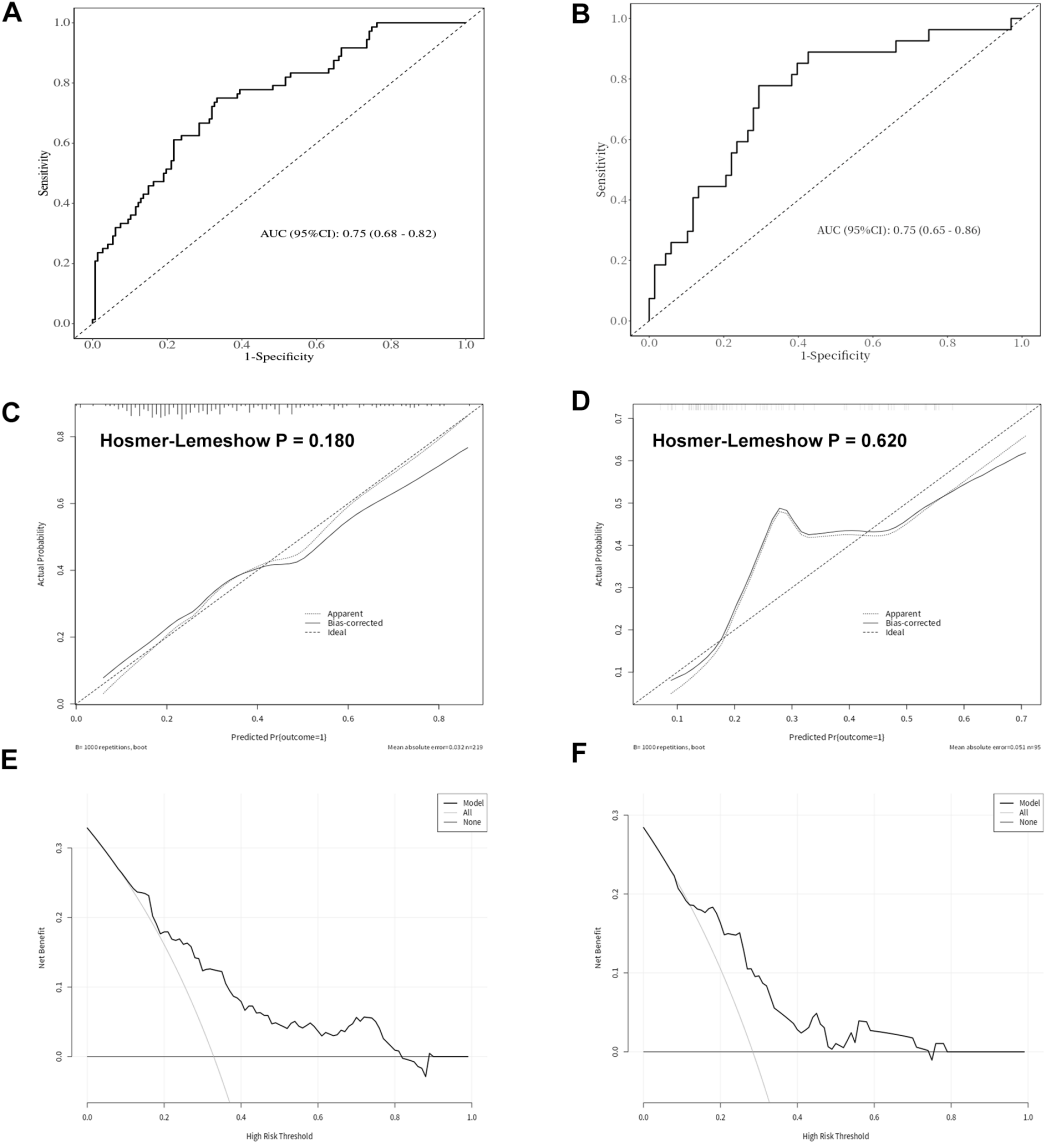
Performance and validation of the nomogram in the training and validation cohorts. **(A)** Receiver operating characteristic (ROC) curve of the nomogram in the training cohort. **(B)** ROC curve of the nomogram in the validation cohort. **(C)** Calibration curve of the nomogram in the training cohort, demonstrating agreement between predicted and observed probabilities. **(D)** Calibration curve of the nomogram in the validation cohort. **(E)** Decision curve analysis (DCA) evaluating the clinical net benefit of the nomogram in the training cohort across a range of threshold probabilities. **(F)** DCA of the nomogram in the validation cohort. The solid line represents the nomogram, while the dashed and horizontal lines represent the treat-all and treat-none strategies, respectively.

We integrated these clinical features to develop a PHI-based model for patients with PSA levels between 4–20 ng/mL, using risk factors including triglycerides, PI-RADS, ALB, and PHI (**Figure 4**). Higher total scores from these risk factors correspond to a higher risk of prostate cancer diagnosis. In the training cohort, the area under the ROC curve (AUC) was 0.75 (0.68–0.82) (**Figure 5A**). Using a prespecified probability threshold of 0.50 (indicating a positive biopsy), the model achieved a sensitivity of 67% and a specificity of 75%. The calibration curve demonstrated good alignment between predicted and observed values, with the Hosmer-Lemeshow test confirming a good fit (P = 0.180) (**Figure 5C**). DCA verified the clinical applicability of the nomogram in the training cohort, demonstrating a favorable net benefit across a wide range of threshold probabilities (**Figure 5E**). In the validation cohort, the AUC was 0.75 (0.65–0.86), with a sensitivity of 62% and specificity of 81% (**Figure 5B**). The calibration curve again showed a good fit, with a *Hosmer-Lemeshow* test result of P = 0.620 (**Figure 5D**). The DCA for the validation cohort also indicated high clinical utility for the predictive model (**Figure 5F**).

## Discussion

The PHI has emerged as an important biomarker in PCa diagnostics, particularly in patients with PSA levels within the diagnostic gray zone (35). PSA alone demonstrates limited specificity and cannot reliably differentiate benign prostatic hyperplasia, prostatitis, and prostate cancer, particularly in men with PSA levels between 4.0 and 20.0 ng/mL (36, 37). In this context, PHI serves as a valuable adjunct biomarker that improves diagnostic accuracy and clinical decision-making.

Accumulating evidence indicates that PHI outperforms PSA alone in predicting prostate cancer and may help reduce unnecessary biopsies while maintaining acceptable sensitivity. A comprehensive meta-analysis by Lusia et al. (38), including 60 studies with sample sizes ranging from 43 to 1,538 patients, evaluated the diagnostic performance of PHI for both overall PCa and csPCa. For PCa, reported sensitivity and specificity ranged from 0.380 to 0.945 and from 0.213 to 0.963, respectively, while for csPCa these ranges were 0.533-1.000 and 0.211-0.885. Considerable heterogeneity across studies was observed, reflecting differences in study design, patient characteristics, definitions of csPCa, and PHI cut-off values.

In our study, PHI remained an independent predictor of prostate cancer in multivariable analysis, reinforcing its diagnostic utility for men with PSA levels ranging from 4 to 20 ng/mL. The relatively modest odds ratio observed for PHI per unit increase is expected, given its continuous nature and wide numeric range. Importantly, small per-unit effect sizes may translate into clinically meaningful differences in risk across the full spectrum of PHI values. Notably, PHI retained statistical significance after adjustment for imaging findings, metabolic parameters, and other clinical variables, underscoring its incremental diagnostic value in patients within the PSA gray zone. For example, Seisen et al. showed that a PHI threshold greater than 40 significantly improved the detection of csPCa and outperformed PCA3 (39). Similarly, a multicenter European study led by Fossati et al. demonstrated that combining p2PSA with PHI improved predictive accuracy for both PCa and csPCa, particularly in younger patients, and that higher PHI values were associated with higher Gleason scores (40). Importantly, their findings suggested that applying a PHI threshold above 29.2 could avoid a substantial number of unnecessary biopsies without missing clinically significant cancers.

The differences between our results and those of previous studies may stem from variations in cohort characteristics, biopsy approaches, sample sizes, and the criteria used to define csPCa. Additionally, variability in PHI cut-off values and methodological differences across studies may influence reported diagnostic performance (41). These factors highlight the need for continued validation of PHI-based models across diverse populations and clinical settings. Notably, our model demonstrated improved discriminatory performance compared with PSA-based approaches alone. By combining PHI with PI-RADS scores from multiparametric MRI, along with triglycerides as a potential metabolic biomarker and albumin as a marker of nutritional and overall health status, the proposed nomogram offers a more comprehensive evaluation of prostate cancer risk. Notably, triglycerides and albumin were identified in our study as independent predictors of prostate cancer. Although both parameters are traditionally regarded as indicators of hepatic function in clinical practice, their association with prostate cancer is more likely to reflect systemic metabolic and inflammatory alterations rather than isolated liver dysfunction. Recent studies have shown that prostate cancer cells exhibit distinct metabolic reprogramming characteristics, particularly manifested as increased lipid uptake, enhanced fatty acid biosynthesis, and active fatty acid oxidation (42). Chronic low-grade inflammation is increasingly recognized as a key contributor to prostate carcinogenesis. Hypoalbuminemia may indicate an inflammatory or catabolic state and has been associated with adverse oncologic outcomes across multiple malignancies (43, 44). It is worth noting that in the multivariate regression analysis, after adjusting for PSA, imaging results and other clinical variables, triglycerides and albumin still maintained statistical significance, suggesting that their predictive value is independent of traditional prostate-specific indicators. Furthermore, this relationship may be partly driven by shared risk factors, including metabolic syndrome, obesity, insulin resistance, and age-related metabolic changes, all of which have been reported to correlate with an increased risk of prostate cancer (45).

Previous studies have shown that PHI-based prediction models outperform PSA alone in differentiating malignant from benign prostate conditions and reduce unnecessary biopsies without compromising sensitivity (46, 47). While noninvasive blood-based markers such as NLR and PLR have also shown potential diagnostic value, their clinical utility remains less well established (13, 14). Compared with previous studies primarily focusing on PSA or imaging parameters alone, the present study provides several noteworthy distinctions. We incorporated metabolic indicators, namely triglycerides and albumin, into a PHI-based predictive framework, rather than relying solely on prostate-specific biomarkers. While most prior models emphasized PSA derivatives or imaging scores, our approach reflects the growing recognition that prostate cancer development is closely intertwined with systemic metabolic and inflammatory alterations. By integrating biochemical, radiological, and metabolic parameters into a unified nomogram, the model offers a more comprehensive assessment of tumor risk, particularly in patients within the PSA gray zone.

Several limitations of this study warrant consideration. First, as a retrospective, single-center investigation, it may be subject to selection bias and its findings may have limited generalizability to broader or more ethnically diverse populations. Second, the overall sample size was relatively modest, particularly the number of patients with csPCa, which may have reduced statistical power. Third, although the model demonstrated improved performance compared with PSA alone, the AUC values indicated only moderate discriminatory ability, suggesting that additional biomarkers or molecular features may further enhance predictive accuracy. Additionally, the study lacked external validation using independent multicenter cohorts. Therefore, the transportability and robustness of the model remain to be confirmed. Finally, although PHI was incorporated as a continuous variable, standardized and population-specific PHI cut-off values were not systematically explored, which may influence clinical implementation.

## Conclusions

The nomogram combining triglycerides, PI-RADS, ALB, and PHI could enhance the detection of PCa in patients with a PSA range of 4–20 ng/mL. Further prospective studies are needed to evaluate the predictive performance of this model across different prostate cancer populations.

## Data Availability

The original contributions presented in the study are included in the article/supplementary material. Further inquiries can be directed to the corresponding authors.
